# The Metabolic Regulatory Mechanisms of Umami Amino Acids in *Stropharia rugosoannulata*

**DOI:** 10.3390/foods15020232

**Published:** 2026-01-08

**Authors:** Mei Wang, Yingyue Shen, Qunli Jin, Lijun Fan, Zuofa Zhang, Ningtao Wei, Xin Huang, Yingmin Qu, Meng Shen, Tingting Song, Weiming Cai

**Affiliations:** Institute of Horticulture, Zhejiang Academy of Agricultural Sciences, Hangzhou 310021, China; wangmei@zaas.ac.cn (M.W.); shenyingyue@zaas.ac.cn (Y.S.); jinql@zaas.ac.cn (Q.J.); fanlj@zaas.ac.cn (L.F.); zhangzuofa@zaas.ac.cn (Z.Z.); w18857493783@163.com (N.W.); hx1624063618@gmail.com (X.H.); 11616057@zju.edu.cn (Y.Q.); nkysm@hotmail.com (M.S.)

**Keywords:** umami flavor, *SrCS*, TCA cycle, glutamic acid, aspartic acid, SrELT1, *S. rugosoannulata*

## Abstract

*Stropharia rugosoannulata* is a widely cultivated edible mushroom known for its nutritional value and umami flavour. Electronic tongue technology and metabolomics revealed that glutamic acid (Glu) and aspartic acid (Asp) levels were positively correlated with umami in the fruiting body developmental stages. Subsequent investigations found that overexpression of *SrCS* within the TCA cycle resulted in decreased levels of Glu and Asp. Integrating TF-gene-metabolite network modelling with experiments identified SrELT1 as a transcriptional regulator of *SrCS*. Different temperatures, cultivation substrates and genetics significantly impacted *SrELT1* and *SrCS* expression, thereby affecting Glu and Asp synthesis. The findings suggest that increased Citrate synthase (CS) activity channelled citrate into glycolysis and oxidative phosphorylation without excessive accumulation; in contrast, decreased CS activity shifted metabolism toward the production of metabolites like Glu and Asp. This study provides insights for enhancing the umami of *S. rugosoannulata*, thereby substantially increasing its market competitiveness in the premium food segment.

## 1. Introduction

*Stropharia rugosoannulata* was initially cultivated in Germany during the 1960s, and was known as saketsubatake in Japanese [1] and *wine-cap Stropharia* in English [1,2]. *S. rugosoannulata* has high nutritional value, a delicious flavour, and is rich in amino acids (AAs), flavonoids, polysaccharides, vitamins, proteins and polyphenolic compounds, which are beneficial to enhancing human immunity [3,4,5,6].

The flavour profile of edible fungi is primarily composed of volatile and non-volatile compounds [7]. Studies indicate that non-volatile components such as free AAs and nucleotides significantly influence the umami profile [7,8]. Primary contributors to umami typically include glutamic acid (Glu), aspartic acid (Asp), inosine, and guanosine [9]. The fruiting body of *S. rugosoannulata* contains 15–21 free amino acids (AAs) at 3.07–9.38% dry weight, with total AA and essential AA content significantly higher than in *Lentinus edodes* [10]. Previous research has found that while AAs such as Glu, glycine (Gly), alanine (Ala), valine (Val), and Asp all contribute to umami taste, Glu and Asp are especially significant [11,12].

The AA content in edible fungi can be influenced by various factors, including cultivation methods, climatic conditions, geographical location and especially temperature [13]. Studies on 13 wild edible Yunnan fungi showed varied AA profiles, highlighting environmental factors’ key role [14]. Soil microbes and nitrogen availability significantly influence AA composition [15], as demonstrated by Ganoderma lucidum’s metabolic responses to nitrogen variation [16].

The biosynthesis of AAs is a complex regulatory process that involves many internal and external factors [17]. Tyrosine (Tyr), tryptophan (Trp) and phenylalanine (Phe) are three aromatic AAs that are biosynthesised through the shikimate pathway [18]. Shikimate dehydrogenase (NADP^+^) catalyses the reversible reduction of 3-dehydroshikimate to shikimate and contributes to AA biosynthesis [19]. Pro-biosynthetic genes, *proABC*s, control proline (Pro) production from Glu, and mutations to the *proA* gene increase Pro production [20]. The expression of *phbCAB* genes in *Corynebacterium glutamicum* was shown to help regulate Glu metabolism [21]. Glutamate synthetase (GS) and glutamine synthetase (GOGAT) are responsible for the assimilation of ammonium into glutamine (Gln) and Glu [22]. Additionally, metabolic studies demonstrated that COP9 signalosome subunit 6 (*CSN6*) expression caused the production of serine (Ser) and Gly [23]. Asparaginase gene *ASPGB1* transcription was associated with soybean seed protein concentration and nitrogen flux [24]. However, amino acid synthesis regulation in *S. rugosoannulata* remains unclear.

This study aims to investigate the metabolome and transcriptome of *S. rugosoannulata* fruiting bodies across three developmental stages using a multi-omics approach. It elucidates amino acid profile changes linked to flavor formation, identifies Glu and Asp as key umami contributors via electronic tongue analyses, and suggests a regulatory mechanism involving the “SrELT1-*SrCS*” module and TCA cycle flux. The research also explores environmental and genetic influences on amino acid synthesis, providing novel insights into transcriptional regulation of umami flavor biosynthesis for application in fungal flavor studies.

## 2. Materials and Methods

### 2.1. Materials and Cultivation Conditions

Two *S. rugosoannulata* strains, MD128 (CGMCC 5.2268) and Zhejiangruixin 19 (CGMCC 41582), were non-commercial strains obtained from the Jiaxing Academy of Agricultural Science in Jiaxing City, Zhejiang Province, China. MD128 is a yellow-pileipellis variant derived from a natural mutation of the reddish-brown strain ZJR 19.

The mycelium was inoculated onto solid substrates formulated with the following primary components: rice straw (RS), mulberry branches (MB), grapevine vines (GV), pectin residue (RP), or mixed wood chips (MWC). These substrates were composed of 83% of the primary ingredient, supplemented by 10% rice husk, 5% bran, 1% lime, and 2% brown sugar. Cultivation was conducted at 25 °C with relative humidity maintained between 65% and 70%, until complete colonisation of the substrate by mycelium was achieved. Subsequently, a layer of casing soil was applied, followed by cold treatment to induce primordium formation and fruiting body development. Fruit bodies were harvested at three distinct developmental stages under identical conditions. In stage II, the pileipellis, pileu and stipe were separated. All samples were frozen rapidly in liquid nitrogen and stored at −80 °C for further analysis. Three biological replicates were used for metabolomic and transcriptomic profiling as well as for downstream experiments.

### 2.2. Electronic Tongue Test Method

Fresh fruiting bodies were aseptically chopped and homogenised, followed by extraction with distilled water 1:5 (*w*/*v*) for 30 min. The homogenate was then centrifuged at 4000 rpm for 10 min, and the supernatant was filtered through a 0.22 μm membrane filter and stored for subsequent analysis. The experiment was performed by Wuhan ProNets Testing Technology Co., Ltd., Wuhan, China. Three biological replicates were prepared for each experimental group. During e-tongue analysis, Insent SA402B was used, a buffer solution at pH 6.0 was used as the reference liquid, and the detection temperature was maintained at 25 ± 1 °C. After injection of each sample into the sensing cell, the electrode response data—including potential changes and impedance signals—were recorded. Principal component analysis (PCA) and linear discriminant analysis (LDA) were employed to quantitatively evaluate the differences in flavour profiles among fruiting bodies at different developmental stages. The data were processed and visualized utilizing the proprietary software integrated with the Insent SA402B system.

### 2.3. Metabolite Extraction and Profiling

Nine samples were used for metabolomic assays performed by Metware Biotechnology (Wuhan, China). Each sample (20 mg) was solubilised in pre-cooled methanol (70% *v*/*v*, 400 μL). The resulting mixture was vortex mixed for 3 min, followed by ultrasonic treatment in an ice-cooled bath for 10 min. Post-sonication, the solution underwent a second vortex agitation (1 min) and was then incubated at 4 °C for 30 min. Following centrifugation, the clarified supernatant was filtered through a membrane and prepared for analytical evaluation.

The analytical platform comprised an ultra-high-performance liquid chromatography (UPLC) system (ExionLC AD, Sciex, Framingham, MA, USA) coupled with a hybrid triple quadrupole-linear ion trap mass spectrometer (QTRAP, Sciex). QC/QA protocol: QC samples (pooled 10 μL of all samples) were injected every 10 samples to monitor stability. L-2-Chlorophenylalanine (10 μg·mL^−1^ final concentration) was used as the internal standard, with 91.2–108.5% recovery. Instrument reproducibility (*n* = 6 consecutive QC injections) produced a peak area RSD < 5.0% and a retention time RSD < 0.3%. Chromatographic separation was achieved using a Waters ACQUITY UPLC HSS T3 C18 column (2.1 mm × 100 mm, 1.8 μm particle size). The mobile phase consisted of two components: (A) aqueous 0.1% formic acid and (B) acetonitrile containing 0.1% formic acid. The UPLC gradient elution program was conducted as follows: starting with 95% water/5% acetonitrile (*v*/*v*) at 0 min, a linear gradient was applied to reach 10% water/90% acetonitrile (*v*/*v*) by 11.0 min, maintaining this composition until 12.0 min, followed by a return to 95% water/5% acetonitrile (*v*/*v*) at 12.1 min and sustaining this until 14.0 min. The analysis was performed at a flow rate of 0.4 mL/min, with a column temperature of 40 °C and an injection volume of 2 μL. The mass spectrometry data were processed by software Analyst 1.6.3. The process involves mixing all sample extracts equally to create QC samples for non-targeted detection on the LC-QTOF-MS/MS platform. Using databases like MWDB, Metlin, HMDB, KEGG, the AI prediction library, and MetDNA, precise qualitative analysis is conducted. Identified metabolites’ multi-ion pairs and retention times are extracted. A new project-specific library is then formed, combining this data with Maiwei’s target database. Finally, metabolites in this new library are precisely quantified in all samples using the Q-Trap instrument platform. Metabolite accumulation patterns were analysed via hierarchical cluster analysis (HCA) in R v3.5.0. Differentially expressed metabolites (DEMs) were identified based on fold change (2 ≤ FC ≤ 0.5) and OPLS-DA VIP ≥ 1, then FDR-corrected using the Benjamini-Hochberg method (FDR < 0.05 as final threshold). DEM pathways were annotated using the KEGG database, and pathway enrichment was assessed through metabolite sets enrichment analysis (MSEA) with *p* ≤ 0.05. DEMs were normalised using unit-variance scaling and analysed using K-means clustering.

### 2.4. Transcriptome Sequencing and Analysis

Nine samples were used for RNA-seq analysis by Wuhan Metware Biotechnology Co., Ltd. (Wuhan, China). Total RNA was extracted using a BioTeke kit (BioTeke, Beijing, China) according to the manufacturer’s instructions. Then, RNA-seq libraries were constructed, PCR-amplified and purified with AMPure XP beads. The libraries were sequenced on the Illumina HiSeq 2000 platform. A total of 71.2 Gb of clean data was collected, with each sample yielding 6 Gb and a Q30 base percentage of at least 94%. The adaptors were removed, and clean data were generated by removing reads < 100 bp from the raw data. The transcriptome sequencing is conducted with a read length of 150 base pairs in both directions. All downstream analyses were based on clean data, which accounted for ≥ 98% of the raw data (6–9.5 Gb per sample), with Phred scores ≥ 30 for ≥94% of bases. This ensured data integrity for subsequent analyses. Base composition analysis showed ≤8% A/T and C/G proportion deviation at each position, without systematic imbalance, ruling out primer residues and sequencer bias. The sequencing depth ranged from 300× to 430×.

The transcriptome was assembled from high-quality reads using Trinity v2.4.0. Local BLAST searches were conducted against non-redundant (nr) protein sequences in the NCBI, Swiss-Prot, COG and KEGG databases for genes. Genome alignment coverage ranged from 93.7% to 95.7%. The FPKM (fragments per kilobase of transcript per million fragments) was used to estimate gene expression levels. DESeq2 (v1.22.1) was used for differential expression analysis between pairs of groups, and the false discovery rate was used to adjust the *p*-values. Genes with significant differences in expression (i.e., log2 fold change > 1 and adjusted *p*-value < 0.01) were considered DEGs and were annotated with GO terms (clusterProfiler v4.6.0).

### 2.5. Transient Overexpression in Tobacco Leaves and Subcellular Localisation Analysis

The full-length coding sequence (ORF) of *SrCS* was amplified from complementary DNA (cDNA) of ZJR19 using primers listed in Table S1 and cloned into the *pCAMBIA1300-sGFP* vector to generate the *35S:SrCS-sGFP* construct. The resulting plasmids were transformed into *Agrobacterium tumefaciens* strain GV3101 and used for infiltration into six-week-old *Nicotiana benthamiana* plants. An empty vector was employed as a negative control. Leaf tissue was harvested five days post-infiltration and stored at −80 °C for subsequent analysis.

Subcellular localisation of SrCS was predicted using WolfPSORT (https://wolfpsort.hgc.jp/). To validate this, the *35S:SrCS-sGFP* construct was transiently expressed in tobacco epidermal cells via Agrobacterium-mediated transformation, utilizing a bacterial culture with an *OD600* value of 1.0. Fluorescence was observed under a confocal laser scanning microscope after 48 h of incubation.

### 2.6. Dual-Luciferase Reporter Assay

To investigate the transcription factors (TFs) that regulated the activity of target genes, TF genes were cloned into the *pGreenII 62-SK* vector to generate effector constructs. The promoter regions of target genes were inserted into the *pGreen II 0800-LUC* vector to produce reporter constructs. The primer sequences of all genes are listed in Table S1. The effector and reporter plasmids were introduced into *A. tumefaciens* strain GV3101, and then co-infiltrated into *N. benthamiana* leaves. An empty *pGreen II 62-SK* vector was used as the negative control.

Luciferase activity was measured using a Dual-Luciferase Reporter Assay System (Promega, Waltham, MA, USA). Relative luciferase activity was calculated as the ratio of firefly luciferase (LUC) to renilla luciferase (REN). All experimental results are presented as the mean ± standard deviation (SD) from three independent biological replicates.

### 2.7. Yeast One-Hybrid Assays

The Clontech Matchmaker One-Hybrid System was employed to conduct yeast one-hybrid assays. The promoter region of *SrCS* was amplified from the genome of ZJR19 using the primers listed in Table S1 and cloned into the *pHIS2* bait vector. cDNA of TFs was inserted into the *pGADT7* prey vector for expression in yeast. Bait and prey constructs were co-transformed into the yeast strain Y187 using the Yeastmaker Yeast Transformation System (Clontech Laboratories, Mountain View, CA, USA). Transformants were selected on SD-TLH medium supplemented with 0, 40 or 50 mM 3-AT to verify successful transformation.

### 2.8. RNA Extraction and Quantitative RT-PCR

Total RNA was isolated from plant tissues using the Easy-Do RNA Extraction Kit (Easydo, Hangzhou, China). Complementary DNA (cDNA) was synthesised from 1 μg of total RNA using the Hifair^®^ II First-Strand cDNA Synthesis Kit (YESEN, Shanghai, China). Quantitative real-time PCR (qRT-PCR) was performed using the Hieff^®^ qPCR SYBR^®^ Green Master Mix (No Rox) (YESEN) according to the manufacturer’s instructions. Relative gene expression levels were calculated using the 2^−ΔΔCT^ method. Primer sequences are listed in Table S1, with 18S rRNA used as an internal reference. Three independent biological replicates were performed for each experiment.

### 2.9. Amino Acid Quantification

Tobacco leaf sample (1 g) was placed in a 25 mL centrifuge tube, mixed with distilled water (5 mL), and sonicated for 30 min. The pH was adjusted to 4, and the volume was brought to 10 mL with ultrapure water. After centrifuging at 5000 rpm for 5 min, the supernatant was filtered through a 0.22 μm aqueous membrane. Amino acid concentrations were then measured by HPLC-MS/MS.

### 2.10. Citrate Quantification

Citrate was quantified by HPLC. Lyophilised sample (0.1 g) was mixed with H_2_SO_4_/H_2_O (0.008 N, 5 mL), shaken for 1 min, and centrifuged at 4000 rpm for 5 min at 4 °C. Supernatant (2 mL) was centrifuged at 12,000 rpm for 2 min at 4 °C. The extract was analysed using HPLC with LC-10AD pumps, an SLC10A system control, a diode array UV-VIS detector and a Synergy Hydro column. Citrate was eluted with H_2_SO_4_/H_2_O (0.008 N) at 1.0 mL·min^−1^ under isocratic conditions, and the absorbance was measured at 210 nm. Each sample was extracted and analysed twice, and results are expressed as mg of organic acids per 1000 mg of fresh matter.

### 2.11. Statistical Analysis

All experiments were conducted with three independent replicates. Data analysis was performed using SPSS (version 24.0), and data visualization was carried out using GraphPad Prism (version 8.0). Prior to conducting parametric tests, the assumptions of normality and homogeneity of variances were evaluated for all datasets using the Shapiro-Wilk test and Levene’s test, respectively. The data satisfied both assumptions (*p* > 0.05), thereby justifying the application of parametric analyses. For comparisons between two independent groups, an unpaired two-tailed Student’s *t*-test was employed. For comparisons involving more than two groups, a one-way analysis of variance (ANOVA) was conducted, followed by Duncan’s post hoc test for multiple comparisons. Data are presented as the mean ± standard deviation (SD) of the replicates, unless otherwise indicated. A *p*-value of less than 0.05 was considered statistically significant in all analyses.

## 3. Results

### 3.1. Taste Dynamics and Metabolomic Profiling of S. rugosoannulata Across Developmental Stages

**Figure 1:**
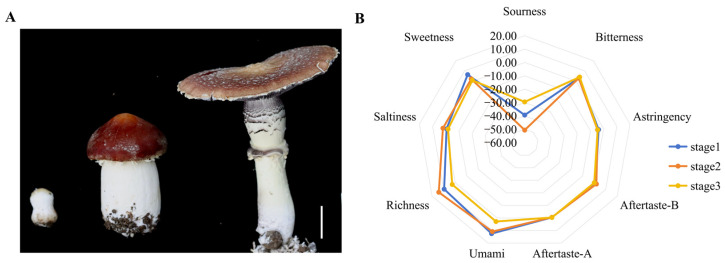
Electronic tongue (E-tongue) analysis of fruiting bodies at different growth stages. (**A**) Photographs of the fruiting body at three stages. Scale bar = 3 cm. (**B**) Radar map of the E-tongue (*n* = 3).

The unique flavour of *S. rugosoannulata* is a key factor in consumer appeal and varies substantially across developmental stages. An electronic tongue was used to detect changes in taste during three developmental stages (Figure 1A,B) (Table S2). The results showed that as the fruiting body matured, its umami and sweetness profiles changed. Notably, during the umbrella-opening stage (The growth stage during which the pileus gradually opens from a closed state to spore maturation), *S. rugosoannulata* exhibited a significant decrease in both umami and sweetness (Figure 1B).

**Figure 2:**
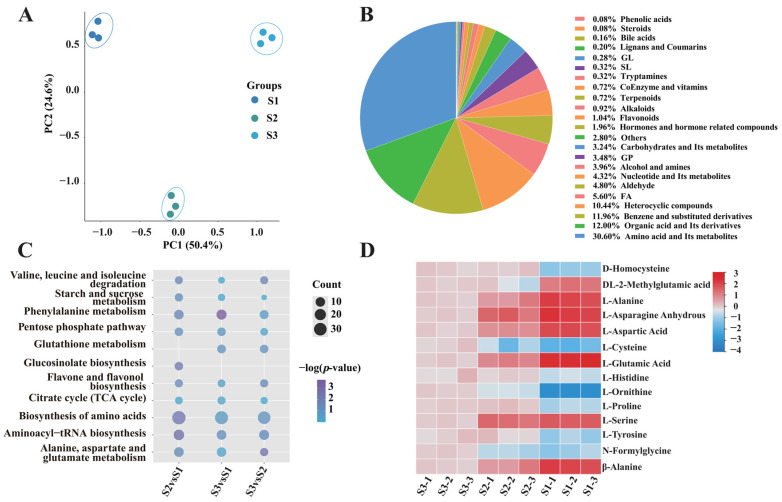
Characterisation of metabolites in the fruiting body of *S. rugosoannulata*. (**A**) PCA of metabolites identified in *S. rugosoannulata*. (**B**) Classification ring diagram of DEMs. (**C**) KEGG pathway enrichment of DEMs in the three developmental stages. The Rich Factor for each pathway is plotted on the *x*-axis, the *y*-axis contains pathway names arranged by *p*-value, the dot colour signifies the *p*-value magnitude (bluer colours denote more significant enrichment), and the dot size corresponds to the count of DEMs. (**D**) Heatmap of the relative AA concentrations in the three developmental stages. The change in colour from red to blue indicates high to low.

To elucidate the metabolite changes in the fruiting body of *S. rugosoannulata*, specimens were collected at three distinct developmental stages: Stage I (bud stage), Stage II (harvesting stage) and Stage III (umbrella stage) (Figure 1A). Nine samples, three from each developmental stage, were analysed using UPLC-MS/MS. PCA of the metabolomic data demonstrated that the samples from the three fruiting body stages could be distinctly separated by the first two principal components, PC1 and PC2, which accounted for 50.4% and 24.6% of the total variance, respectively (Figure 2A). A total of 2501 metabolites were identified, which were categorised into 23 classes, including AAs and their metabolites (30.6%), and organic acids and their derivatives (12%) (Figure 2B). Venn diagram analysis indicated a substantial overlap of DEMs among the comparisons, with 309, 330 and 143 DEMs identified in the S2 vs. S1, S3 vs. S1 and S3 vs. S2 comparisons, respectively (Figure S1). Furthermore, 176, 142 and 103 unique DEMs were detected in the S2 vs. S1, S3 vs. S1 and S3 vs. S2 comparisons, respectively (Figure S1).

Next, KEGG database-based DEM analysis was performed to identify metabolic pathway enrichment. The data were screened according to their *p*-values, and the top 20 significantly enriched pathways were selected for analysis. These pathways included Biosynthesis of AAs; Asp and Glu metabolism; TCA cycle; Starch and sucrose metabolism, and Pentose phosphate pathway (Figure 2C). To elucidate fruiting-body development dynamics, k-means clustering analysis was employed to categorize differential accumulated metabolites (DAMs) into six distinct clusters (CI–CVI) based on their accumulation patterns. Cluster CI exhibited peak metabolite accumulation in the S2 stage, with higher levels in S1 than S3, involving Val, Leu, Ile biosynthesis, Phe metabolism, and Arg pathways. Cluster CII showed declining metabolites during development, linked to Ala, Asp, Glu metabolism, energy pathways, and the TCA cycle. Cluster CIII displayed increasing metabolites, enriched in Phe, Tyr, Trp biosynthesis and Arg/Pro metabolism. Cluster CIV showed minimal S1–S2 changes but significant S3 elevation, associated with galactose and purine metabolism. Cluster CV revealed decreasing metabolites from S1–S2, followed by partial recovery in S3, involving lipid and nitrogen metabolism. Cluster CVI peaked in S2, with reduced S1 accumulation compared to S3, linked to one-carbon pool and folate metabolism (Figure S2). These metabolic trajectories likely regulate biochemical changes, particularly flavor and nutritional compounds, during fruiting-body development.

On this basis, the accumulation and variation in Aas were further analysed. The results revealed that the content of 14 AAs in the *S. rugosoannulata* fruiting body varied greatly in stages I–III (Figure 2D). A notable decrease in the concentrations of Asn, Asp and Glu was observed from stage I to stage III, aligning with the documented changes in umami profiles of *S. rugosoannulata* (Figure 1B).

### 3.2. Effects of Genetic Background and Environmental Factors on L-Asp and L-Glu Concentrations in S. rugosoannulata

**Figure 3:**
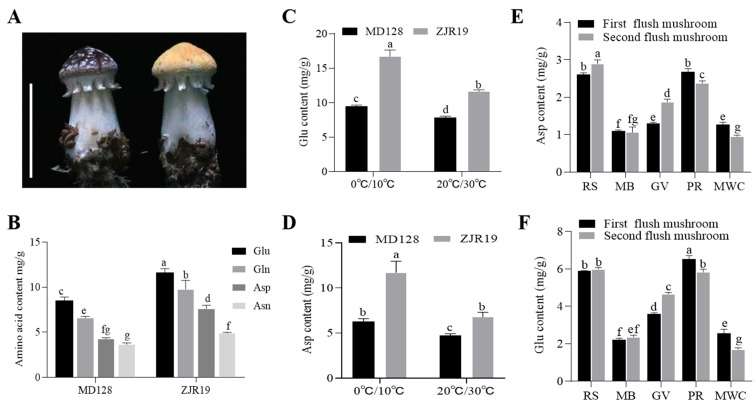
Effects of genetic background and environmental variables on the levels of Asp and Glu in *S. rugosoannulata*. (**A**) Fruiting bodies of the MD128 (wild) and ZJR19 (mutant) strains; scale bar = 3 cm. (**B**) Levels of umami AAs (Asn, Asp, Glu and Gln) among the strains of *S. rugosoannulata*. (**C**,**D**) Content of Glu and Asp in the fruiting bodies of *S. rugosoannulata* under low-temperature treatment. Temperature control for fruiting body growth: 0 °C/10 °C means 10 °C during the day and 0 °C at night; 20 °C/30 °C means 30 °C during the day and 20 °C at night. (**E**,**F**) Comparison of Asp and Glu content in fruiting bodies cultivated under different substrate formulations (RS, rice straw; MB, mulberry branches; GV, grape vines; PR, pectin residue; MWC, mixed wood chips). Data in (**B**–**F**) are represented as mean ± SD (*n* = 3). Different lowercase letters indicate significant differences in each group (Duncan’s test, *p* < 0.05).

Asp and Glu are crucial AAs for umami flavour. To further investigate their variability patterns, Asp and Glu levels in the MD128 strain (the wild strain) and the ZJR19 strain (the mutant strain) were determined (Figure 3A). The results indicated significant changes in umami AAs—including Asn, Asp, Glu and Gln—between the different varieties of *S. rugosoannulata*. The content of umami AAs in ZJR19 was significantly higher than in MD128, with higher Glu than Asp content (Figure 3B).

Previous studies have demonstrated that temperature influences the umami taste of mushrooms. Accordingly, a low-temperature treatment during the fruiting period of *S. rugosoannulata* was implemented, and the concentrations of Glu and Asp in the fruiting bodies were subsequently measured. The results indicated that, relative to normal temperature conditions, the levels of Glu and Asp were significantly elevated under low-temperature treatment (Figure 3C,D). Furthermore, the Asp and Glu content in the MD128 strain was notably lower than that in the ZJR19 strain.

Selecting appropriate substrates for mushroom cultivation is crucial for increasing yield and improving texture. Studies have shown that different substrate combinations can significantly affect the growth characteristics and nutritional components of mushrooms [25]. In this study, five distinct substrates were employed for the cultivation of *S. rugosoannulata*: rice straw (RS), mulberry branches (MB), grape vines (GV), pectin residue (PR) and mixed wood chips (MWC). Subsequently, the concentrations of Asp and Glu in the fruiting bodies of the mushrooms grown on each substrate were analysed. The findings indicated that *S. rugosoannulata* cultivated on RS and PR exhibited the highest levels of Asp and Glu, whereas those grown on MB and MWC demonstrated the lowest concentrations (Figure 3E,F).

### 3.3. Expression Dynamics of SrCS and Its Influence on Asp and Glu Content in S. rugosoannulata

**Figure 4:**
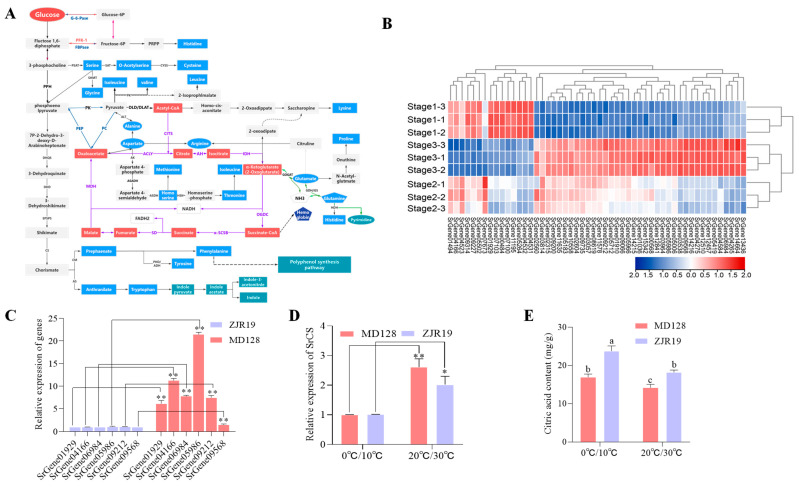
Biosynthesis pathway of AAs that involve *SrCS* in *S. rugosoannulata*. (**A**) Schematic diagram of AA biosynthesis pathways in *S. rugosoannulata*. (**B**) Analysis of differentially expressed genes involved in AA biosynthesis pathways. The change in colour from red to blue indicates high to low. (**C**) Expression of six key candidate genes in the varieties of *S. rugosoannulata*. (**D**) Analysis of *SrCS* expression in the two varieties of *S. rugosoannulata* under different temperature treatments. * means *p* < 0.05, ** means *p* < 0.01 (Student’s *t* test). (**E**) citrate content in fruiting bodies grown under different temperature treatments. The data in (**C**–**E**) are shown as mean ± SD (*n* = 3). Significant differences within each group are marked by different lowercase letters (Duncan test, *p* < 0.05).

Amino acids are synthesised from glucose via glycolysis and the TCA cycle [26]. In the previous KEGG analysis, enrichment in the TCA cycle, starch and sucrose metabolism, and the pentose phosphate pathway was observed (Figure 2C). Thus, the role of the TCA cycle in AA biosynthesis in *S. rugosoannulata* was investigated further. Based on evidence from other species and data from the transcriptome and metabolome of *S. rugosoannulata*, the biosynthetic pathway of AAs in *S. rugosoannulata* was proposed (Figure 4A). In total, 52 unigenes encoding 44 enzymes from the transcriptome and 22 AAs from the metabolome involved in AA synthesis were identified (Figure 4B) (Table S3). Pearson’s correlation analysis was employed to investigate the relationship between candidate gene expression levels and amino acids (AAs) that exhibited significant variations in concentration across the three developmental stages, with an absolute R-value exceeding 0.8. The results showed that *SrGene05986* and *SrGene06984*, encoding citrate synthase (*SrCS*), *SrGene09568*, encoding ATP citrate (pro-S)-lyase (*SrACLY*), *SrGene09212*, encoding pyruvate carboxylase (*SrPC*), *SrGene04166*, encoding fumarate hydratase (*SrfumC*) and *SrGene01929*, encoding malate dehydrogenase (*SrMDH2*)—which are involved in the TCA cycle—were positively and significantly correlated with at least 10 Aas, and significantly negatively correlated with L-Asp and L-Glu (Figure S3).

Expression analysis of pivotal genes in the TCA cycle revealed that the levels of these six candidate genes were elevated in MD128 compared to ZJR19, with *SrGene05986* exhibiting a 21.5-fold increase in expression (Figure 4C). The coding sequences of *SrGene05986,* named *SrCS*, were cloned and selected for further study. CS is the rate-limiting enzyme in the TCA cycle, and oxaloacetate (OAA) and acetyl-coenzyme A (A-CoA) are converted into citrate via the Citrate synthase reaction [27]. Under low-temperature treatment, *SrCS* expression in both varieties decreased significantly (Figure 4D). However, citrate content increased significantly in the fruiting bodies grown at low temperature (Figure 4E). Similarly, the citrate content decreased significantly during fruiting body growth and development (Figure S4).

The expression of *SrCS* in fruiting bodies cultivated with different substrate formulations was further analysed. Similarly, the expression of *SrCS* in the rice straw and pectin residue groups, which had high yields of Asp and Glu, was significantly lower than that in the mulberry branch and grape branch groups. This indicated that the expression of *SrCS* was negatively correlated with Asp and Glu content.

### 3.4. Role of SrCS in Mitochondrial TCA Cycle and Amino Acid Synthesis

**Figure 5:**
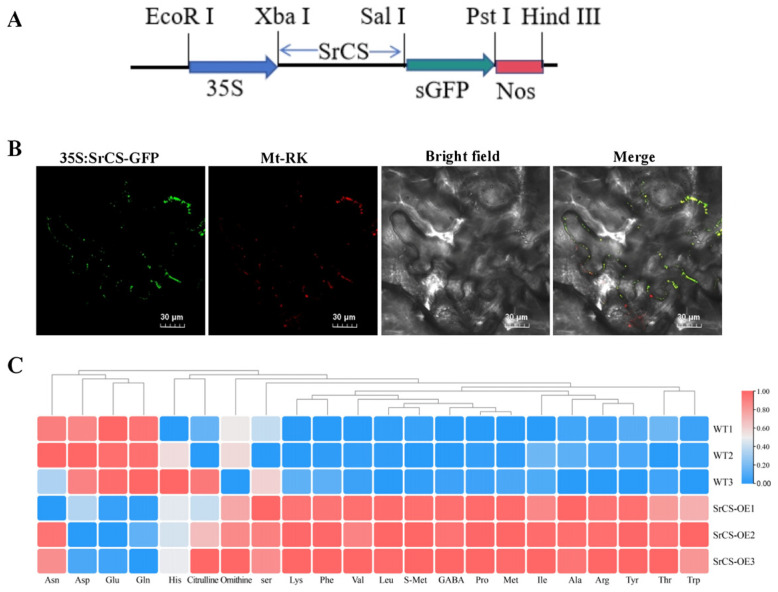
Function of SrCS in the mitochondrial TCA cycle and AA synthesis. (**A**) Constructed expression vector with full-length CDS of *SrCS* inserted into *pCAMBIA1300*. (**B**) Subcellular localisation of SrCS using *35S:SrCS-sGFP* fusion plasmids, as well as mitochondrial marker, transiently expressed in *N. benthamiana* leaves. Scale bar = 30 µm. (**C**) Heatmap of AA content in the *OE-SrCS* strain and wild type. The change in colour from red to blue indicates high to low AA content. Values are expressed as mean ± SD, *n* = 3.

To investigate the subcellular localisation of SrCS, a *35S:SrCS-GFP* fusion plasmid was constructed and transiently expressed into tobacco leaves. Confocal microscopy indicated that the strong green fluorescent protein (GFP) signals of SrCS were co-localised with the mCherry signals of mitochondria (Mt-RK), suggesting that SrCS was localised to the mitochondria (Figure 5A,B). This result indicated that SrCS was associated with the mitochondrial TCA cycle. Next, the *SrCS* overexpression vectors were transformed into tobacco and the AA content of the tobacco was determined using HPLC-MS. The results indicated that the overexpression of *SrCS* led to a significant increase in the content of most AAs compared to the wild type (WT). Specifically, the content of Leu, Ile, Ala, Pro, Val, Thr, Lys, Met, Phe, Arg, Tyr and Trp was significantly higher in *OE-SrCS* compared to the WT. Conversely, the content of Asp, Glu and Gln was significantly lower in *OE-SrCS* compared to the WT (Figure 5C), which was consistent with the results of the metabolic transcriptional association analysis. This finding substantiated that SrCS plays an important role in AA biosynthesis in *S. rugosoannulata*, specifically contributing to the synthesis of umami AAs, which were subject to negative regulation.

### 3.5. Activation of SrCS Promoters by GATA-like Transcription Factor SrELT1 in Amino Acid Biosynthesis

**Figure 6:**
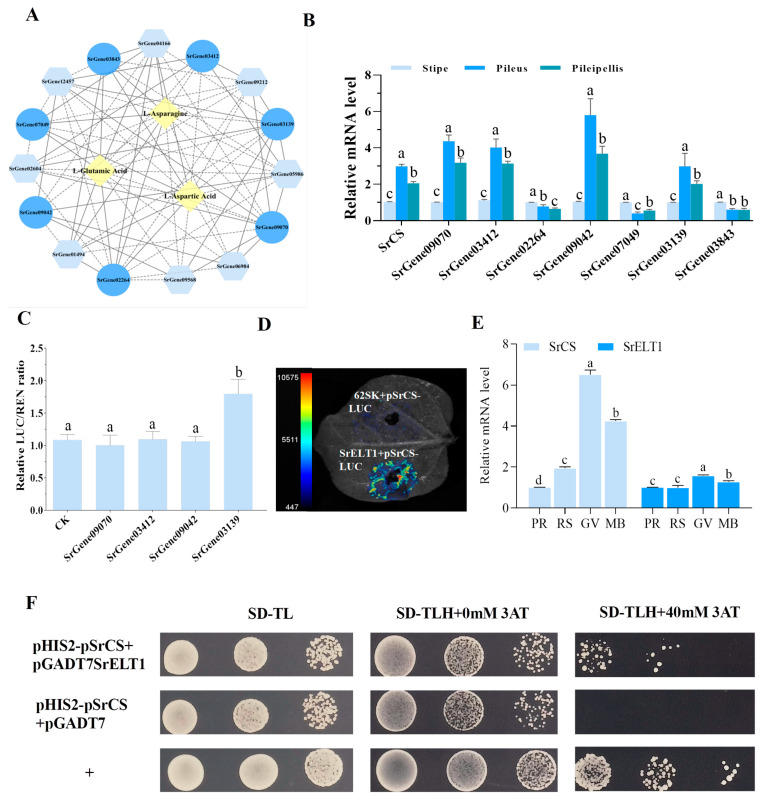
SrELT1 modulates amino acid biosynthesis via *SrCS* regulation in *S. rugosoannulata*. (**A**) Interaction network of TFs-gene expression-metabolites related to AA synthetic pathways. Circles represent TFs, rhombuses represent metabolites and hexagons represent structural genes. Solid and dotted lines represent positive and negative correlations, respectively, with Pearson’s correlation coefficient ABS ≥ 0.80. (**B**) Expression levels of *SrCS* and its associated TFs from the network in the pileipellis, pileus and stipe of *S. rugosoannulata*, and (**C**) effect of four candidate TFs on *SrCS* promoter activity as determined by dual-molecule luciferase experiments. (**D**) Imaging of luciferase enzyme activity of *SrCS* promoters. Luciferase activity was detected in the tobacco leaves. The *62SK* construct was used as a negative control. (**E**) Expression analysis of *SrELT1* and *SrCS* in fruiting bodies cultivated under different substrate formulations. The data in (**B**,**C**,**E**) are represented as mean ± SD (*n* = 3). Different lowercase letters indicate significant differences (Duncan’s test, *p* < 0.05). (**F**) Yeast one-hybrid assays for the interaction between *SrELT1* and *SrCS* promoters.

To identify the significant TFs that were possibly driving changes in AA content, including Asp, Asn and Glu, a network of TFs-gene expression-metabolites with Pearson correlation coefficients ≥ 0.80 was constructed (Figure 6A). In this network, the expression levels of *SrCS* and its associated TFs were investigated in the pileipellis, pileus and stipe of *S. rugosoannulata* using qRT-PCR. The results suggested that *SrCS*, *SrGene09070*, *SrGene03412*, *SrGene09042* and *SrGene03139* were coexpressed in multiple tissues and had the highest levels of coexpression in the pileus (Figure 6B). Based on this, a dual-luciferase reporter system conducted to identify the TFs that could activate *SrCS* promoters. The results showed that only *SrGene03139*, which encodes GATA-like TF SrELT1, could activate the expression of *SrCS* in AA biosynthesis (Figure 6C,D). One hybrid assay was performed, revealing that SrELT1 directly interacted with the *SrCS* promoter (Figure 6F). Furthermore, the expression of *SrELT1* and *SrCS* was synergistically regulated in different substrate formulations and developmental stages of the fruiting body, indicating that SrELT1 may influence Asp and Glu synthesis by regulating *SrCS* expression (Figure 6E and Figure S5).

## 4. Discussion

### 4.1. Role of Asp and Glu in S. rugosoannulata Flavour Evolution

Flavor and nutrition are critical quality indicators for edible mushrooms [28]. Electronic tongues analyze flavor profiles, differentiating taste variations during growth stages [29]. Analysis reveals reduced umami in mature *S. rugosoannulata* (Figure 1B). Umami in edible fungi stems from umami AAs and 5′-nucleotides [30], with Glu, and Asp as key contributors [31]. Accordingly, high-quality metabolic data from different developmental stages of *S. rugosoannulata* were obtained, and metabolomic analysis demonstrated a significant enrichment in the biosynthesis of AAs, with pronounced alterations in Asp and Glu (Figure 2B,C). Earlier research has indicated that although Glu, Gly, Ala, Val and Asp all play a role in umami flavour, Glu and Asp are particularly significant [32]. Notably, the accumulation of Asp and Glu decreased markedly as the fruiting bodies matured (Figure 2D). This indicates that Asp and Glu are integral to the development of umami flavour in the fruiting body of *S. rugosoannulata*, which can inform timely harvesting.

### 4.2. Influence of Genetic and Environmental Factors on Umami Amino Acid Content in S. rugosoannulata

Umami AA are recognised for their role in flavour formation, which is affected by both genetic and environmental factors [33,34]. The variations in Asp and Glu content within the fruiting body of *S. rugosoannulata* under varying temperature conditions and substrate formulations were investigated. The findings indicated substantial differences in Asp and Glu content. Lower temperatures facilitated the accumulation of Asp and Glu (Figure 3C,D), while the utilisation of straw and pectin residue–based formulations markedly enhanced the Asp and Glu content in the fruiting bodies (Figure 3E,F). These findings were consistent with those of a previous multi-omics analysis of *Pleurotus ostreatus*, in which low-temperature fruiting led to significant increases in AA content, particularly arginine. Additionally, pH changes under low-temperature conditions were closely linked to alterations in citrate and arginine levels [35]. Furthermore, prior studies have indicated that rice straw enhances the content of monosodium glutamate (MSG)–like AAs in mushrooms, with the equivalent umami concentration (EUC) value increasing with the addition of rice straw [36]. This suggests that the cultivation environment influences flavour formation by regulating metabolic processes. However, these findings were primarily at the physiological phenotypes level; data regarding internal regulatory pathways are lacking.

Comparative analysis of different *S. rugosoannulata* varieties ZJR19 and MD128 revealed significant differences in the content of umami AAs, with ZJR19 exhibiting significantly higher levels than MD128 (Figure 3A,B). This directly underscored the pivotal role of genetic background in determining the fundamental synthetic capacity of *S. rugosoannulata* for Asp and Glu. In conclusion, the content of umami AAs in *S. rugosoannulata* was influenced by both genetic and environmental factors.

### 4.3. SrCS-Mediated Carbon Flux Allocation Governs Umami Amino Acid Synthesis

Although numerous metabolites associated with edible mushroom quality have been identified, the metabolic pathways and regulatory networks underlying umami AAs accumulation remain incompletely understood. To address this gap, integrated metabolomic and transcriptomic analyses were performed across three developmental stages of *S. rugosoannulata*, enabling the construction of a regulatory network for AAs metabolism (Figure 4A). This approach revealed coordinated remodeling of central carbon metabolism and amino acid synthesis during fruiting body development.

Glucose-derived carbon enters cellular metabolism through glycolysis, generating pyruvate and ATP, and is subsequently funneled into the TCA cycle, which supplies both energy and key precursors for AA biosynthesis [9,26]. Correlation analysis identified six TCA cycle–associated genes significantly linked to AA profiles (Figure S3). Among these, *SrCS*, encoding citrate synthase, emerged as the gene most strongly associated with Asp and Glu metabolism across multiple cultivation conditions, including different varieties, temperatures, and substrates (Figure 4D,E). SrCS catalyzes the first committed step of the TCA cycle by condensing acetyl-CoA and oxaloacetate to form citrate, thereby controlling carbon entry into respiratory metabolism [37,38]. Subcellular localization confirmed that SrCS is targeted to mitochondria (Figure 5B), consistent with its conserved role in eukaryotic energy metabolism [39].

Functional validation further demonstrated the regulatory role of SrCS in umami AAs accumulation. Overexpression of *SrCS* in tobacco increased total AAs content but led to a marked reduction in Asp, Glu, and Gln levels. This pattern was consistent with Pearson correlation analysis between AA metabolites and candidate genes (Figure 5C), indicating that elevated citrate synthase activity negatively regulates Asp and Glu accumulation. A notable limitation of this study is the absence of *S. rugosoannulata* knockout mutants, attributable to the lack of a genetic transformation system, which constrains direct functional validation within the native fungal context. To circumvent this limitation, a heterologous expression system in tobacco was utilized, a common strategy for investigating fungal gene function when native transformation is not feasible [40]. Although this system does not replicate the full genomic and regulatory complexity of *S. rugosoannulata*, the conserved nature of the TCA cycle and AA biosynthesis pathways across eukaryotes allowed for meaningful insights into the regulatory role of *SrCS*. Future research endeavors should prioritize the development of genetic tools for *S. rugosoannulata* to enhance the precision and reliability of these conclusions.

Metabolic profiling revealed that citrate content varied dynamically across developmental stages and environmental conditions. Notably, citrate levels declined in mature fruiting bodies but increased under low-temperature conditions, trends that closely paralleled changes in Asp and Glu abundance (Figure 3C,D and Figure 4E). However, these patterns were not always aligned with *SrCS* transcript levels (Figure 4D), indicating that citrate accumulation and downstream AAs synthesis are primarily governed by metabolic flux regulation rather than transcriptional control alone.

**Figure 7:**
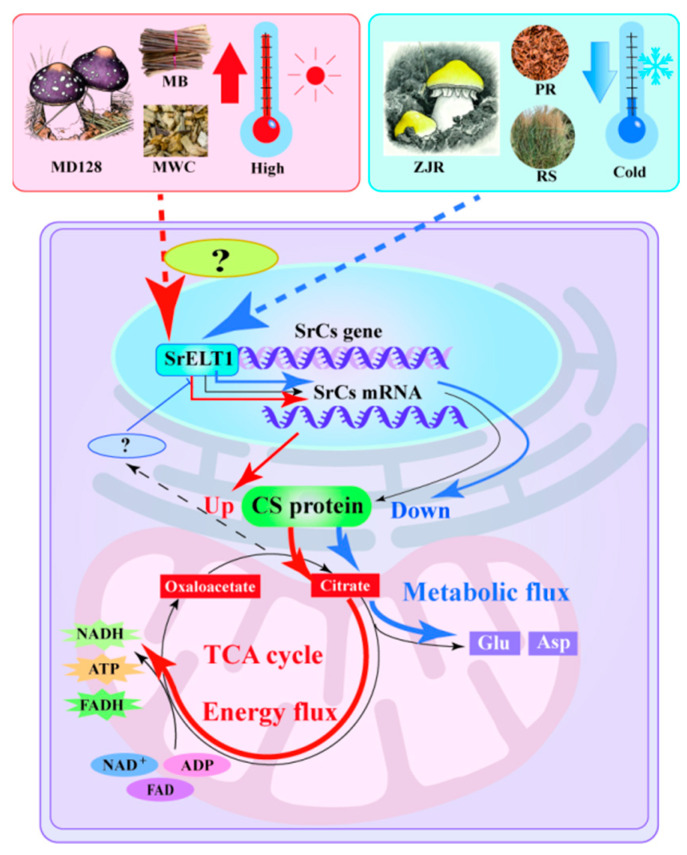
Schematic diagram representing the dynamics of umami amino acid metabolism changes and regulatory mechanisms during umami formation by *S. rugosoannulata*. Red arrows indicate enhanced CS protein accumulation, redirecting energy flow towards ATP synthesis, while blue arrows represent decreased SrCS expression coupled with increased Asp and Glu accumulation.

Substrate-dependent differences provided further mechanistic insight. Fruiting bodies cultivated on lignin-rich substrates (grapevine and mulberry branches) exhibited higher *SrCS* expression but significantly lower Asp and Glu contents compared with those grown on cellulose- and sugar-rich substrates such as rice straw and pectin residue (Figure 3E,F). Lignin degradation imposes high demands for ATP and reducing power, relying on cytochrome P450 monooxygenases and oxidative enzymes such as laccases that consume NAD(P)H [41,42,43,44,45]. Under these conditions, elevated *SrCS* expression enhances TCA cycle flux, accelerating acetyl-CoA oxidation and promoting the generation of NADH and FADH_2_ to support oxidative phosphorylation and energy-intensive lignin breakdown (Figure 7). As a consequence, TCA intermediates are rapidly consumed, limiting their availability for AAs biosynthesis. In contrast, substrates rich in readily metabolizable carbohydrates provide sufficient energy through glycolysis, reducing reliance on an accelerated TCA cycle [46]. Lower *SrCS* expression under these conditions results in reduced TCA flux and promotes the accumulation of oxaloacetate and α-ketoglutarate [47]. These intermediates are preferentially diverted into umami AAs biosynthesis: oxaloacetate is converted to Asp via transamination, while α-ketoglutarate is converted to Glu through glutamate dehydrogenase (GDH)–mediated reactions [48,49]. Thus, low SrCS activity favors carbon allocation toward Asp and Glu synthesis, whereas high SrCS activity channels carbon toward complete oxidation for ATP generation.

Collectively, these findings identify SrCS as a metabolic switch that balances carbon flux between energy production and umami amino acid biosynthesis. This flux-based regulatory mechanism provides a clear explanation for substrate-dependent flavor formation in *S. rugosoannulata* and offers practical guidance for optimizing cultivation strategies to enhance umami quality through targeted substrate design and metabolic regulation (Figure 7).

### 4.4. Exploring the Role of GATA-like Transcription Factor SrELT1 in TCA Cycle Regulation

Although homologous *SrCS* genes have been identified in various plant species, the transcriptional regulation of *SrCS* remains poorly understood. Accordingly, a regulatory network of TFs-gene expression-Asp and Glu metabolic was constructed. The upstream transcriptional regulator of *SrCS*, SrELT1, was screened and validated (Figure 6). ELT1, a GATA-like TF, plays a crucial role in the differentiation and upkeep of hypodermal seam cells, as well as in the regulation of locomotion in *Caenorhabditis elegans* [50]. The present study revealed, for the first time, significant cooperativity between the expression pattern of *SrELT1* and CS activity across different developmental stages of the fruiting body of *S. rugosoannulata,* and under various substrate conditions (Figure 6E). Furthermore, the expression levels of both *SrELT1* and *CS* showed a marked negative correlation with the accumulation of citrate and glutamate (Figure 3E,F). This inverse relationship strongly suggests that SrELT1 may function by upregulating *SrCS* expression, thereby promoting the flux of citrate toward downstream metabolic pathways for ATP production and enhancing TCA cycle activity, thereby reducing the accumulation of its terminal metabolites (Figure 7).

Additionally, the accumulation of citrate may affect the expression regulation of downstream SrCS by TF SrELT1, thereby maintaining the dynamic balance of metabolic flow (Figure 7). However, the specific mechanism underlying the relationship between SrELT1 and AAs remains largely unknown, necessitating further research. These findings provide a novel perspective on the precise regulation of the TCA cycle in fungal developmental metabolism.

## 5. Conclusions

This study demonstrated that Asp and Glu levels, along with umami flavour, decreased synergistically during fruiting body maturation, underscoring their essential role in *S. rugosoannulata* flavour development. By integrating metabolic and transcriptomic data, the *SrCS* gene—which encodes citrate synthase—was identified as a critical regulator of AA synthesis via the TCA cycle. Overexpression of *SrCS* in tobacco demonstrated its negative regulatory impact on Asp and Glu. Lower temperatures and straw-based substrates decreased *SrCS* expression and increased Asp and Glu accumulation, with genetic differences among strains also playing a significant role. Further investigations revealed that increased SrCS activity did not result in citrate overaccumulation; rather, it facilitated metabolic flux reprogramming, enzyme feedback regulation and metabolite recycling, thereby directing citrate into downstream pathways to maintain energy homeostasis and support biosynthetic processes. Additionally, SrELT1, a GATA-like TF, was identified as a regulator of *SrCS*, contributing to the maintenance of metabolic homeostasis. These findings provide critical targets for breeding improvement, industrial fermentation optimization, and cultivation technology upgrades.

## Data Availability

The original contributions presented in the study are included in the article/Supplementary Materials, further inquiries can be directed to the corresponding authors.

## Supplementary Materials

The following supporting information can be downloaded at: https://www.mdpi.com/article/10.3390/foods15020232/s1, Figure S1: Wayne diagram of DEMs in the three groups. Figure S2: Metabolite subclass (GSC) analysis by k-mer among the three stages. Figure S3: Pearson correlation analysis between AA coding genes and metabolites in AA synthesis pathways. Figure S4: citrate content in fruiting bodies at different growth stages. Figure S5: Expression analysis of *SrELT1* and *SrCS* in fruiting bodies at different growth stages. Table S1: Primers used in this study. Table S2: The electronic tongue measurement values. Table S3: DEMs and genes involved in AA synthesis.
