# Supplementary material for: The Metabolic Regulatory Mechanisms of Umami Amino Acids in Stropharia rugosoannulata
Source: Foods. 2026 Jan 8;15(2):232. doi: 10.3390/foods15020232 (PMC12839930; doi:10.3390/foods15020232)
Supplement: Supplementary file 1 [file foods-15-00232-s001.zip › Supplementary.pdf]

Table S1. Primers used in this study.

Table S2. Differential metabolites and genes involved in amino acids synthesis.

Figure S1 Wayne diagram of differential metabolites in three groups.

Figure S2 Metabolites subclasses (GSCs) analysis by k-mer among the three stages samples.

Figure S3 Pearson correlation analysis between amino acid coding genes and metabolites in amino acid synthesis pathway. The significant ones are marked with * ($p < 0.05$).

Figure S4 Citric acid content in fruiting bodies at different growth stages. The data are shown as mean±SD (n = 3). Significant differences within each group are marked by different lowercase letters (Duncan test, $P < 0.05$).

Figure S5 Expression analysis of *SrELT1* and *SrCS* in fruiting bodies at different growth stages. The mean ± SD (n = 3) represents the data. Significant differences are indicated by varying lowercase letters (Duncan test, $P < 0.05$).

(Figure S1)

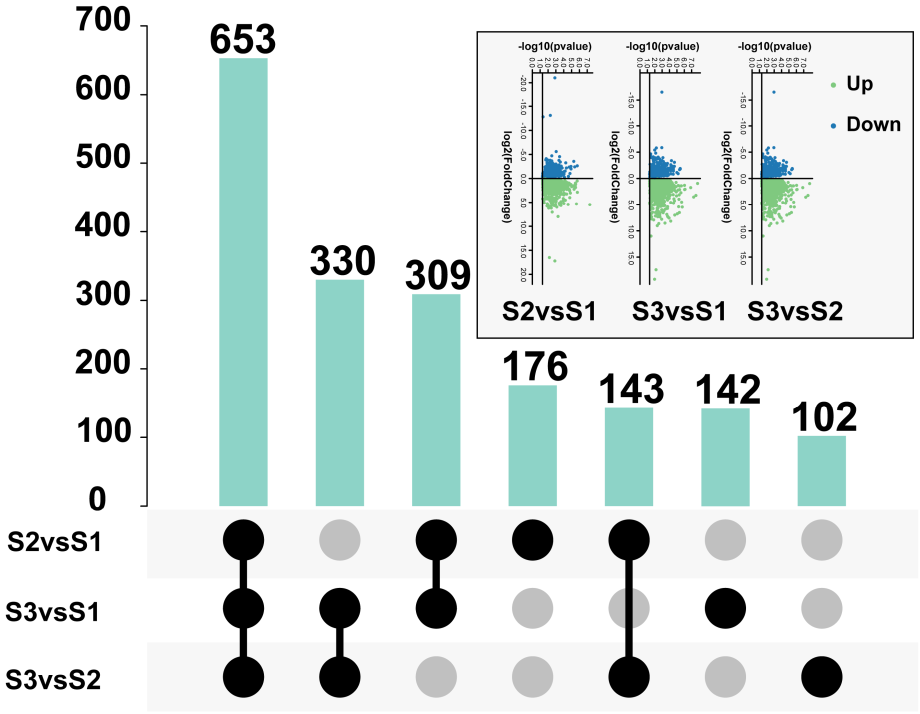

700
600
500
400
300
200
100
0
653
330
309
176
143
142
102
S2vsS1
S3vsS1
S3vsS2
-log10(pvalue)
log2(FoldChange)
Up
Down
S2vsS1
S3vsS1
S3vsS2

(Figure S2)

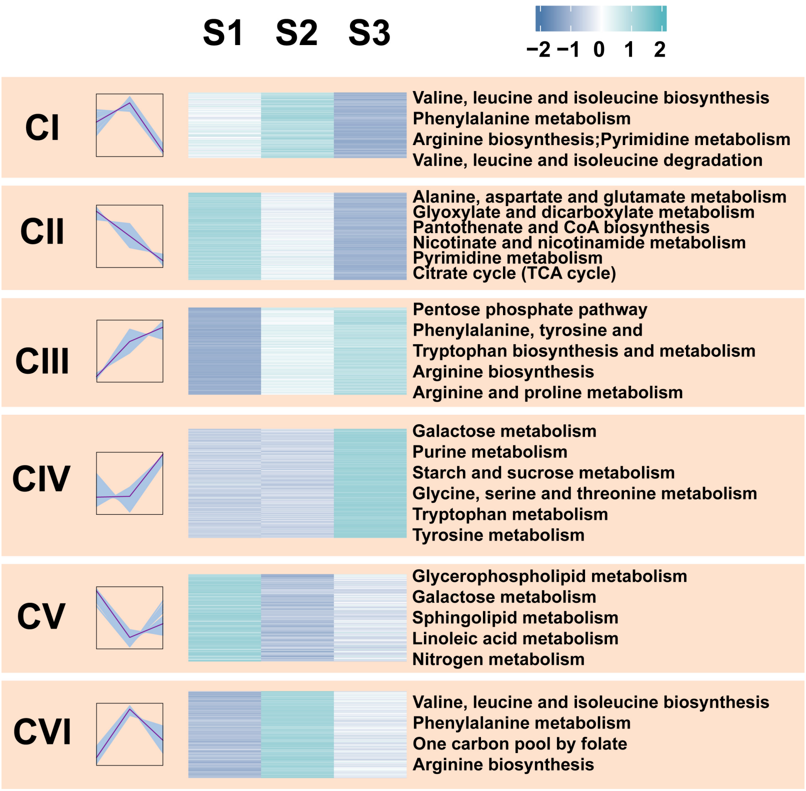
S1 S2 S3
−2 −1 0 1 2
CI
Valine, leucine and isoleucine biosynthesis
Phenylalanine metabolism
Arginine biosynthesis;Pyrimidine metabolism
Valine, leucine and isoleucine degradation
CII
Alanine, aspartate and glutamate metabolism
Glyoxylate and dicarboxylate metabolism
Pantothenate and CoA biosynthesis
Nicotinate and nicotinamide metabolism
Pyrimidine metabolism
Citrate cycle (TCA cycle)
CIII
Pentose phosphate pathway
Phenylalanine, tyrosine and
Tryptophan biosynthesis and metabolism
Arginine biosynthesis
Arginine and proline metabolism
CIV
Galactose metabolism
Purine metabolism
Starch and sucrose metabolism
Glycine, serine and threonine metabolism
Tryptophan metabolism
Tyrosine metabolism
CV
Glycerophospholipid metabolism
Galactose metabolism
Sphingolipid metabolism
Linoleic acid metabolism
Nitrogen metabolism
CVI
Valine, leucine and isoleucine biosynthesis
Phenylalanine metabolism
One carbon pool by folate
Arginine biosynthesis

(Figure S3)

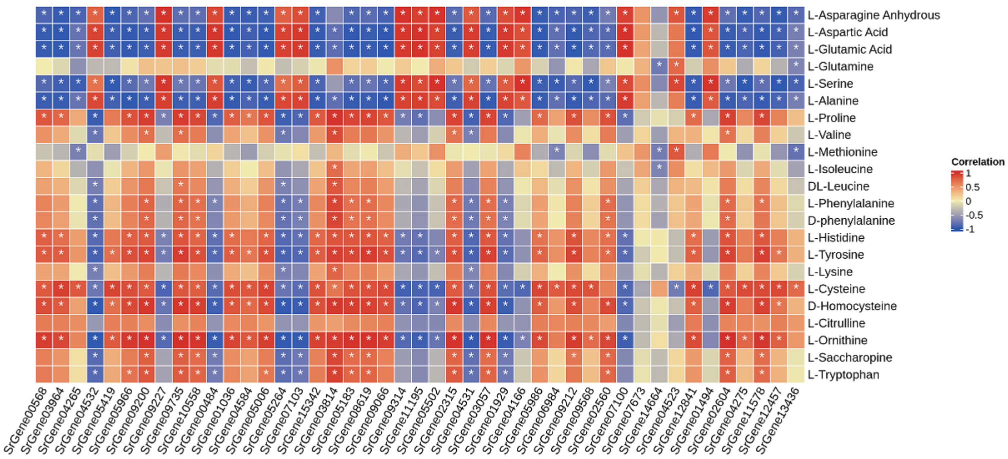
L-Asparagine Anhydrous
L-Aspartic Acid
L-Glutamic Acid
L-Glutamine
L-Serine
L-Alanine
L-Proline
L-Valine
L-Methionine
L-Isoleucine
DL-Leucine
L-Phenylalanine
D-phenylalanine
L-Histidine
L-Tyrosine
L-Lysine
L-Cysteine
D-Homocysteine
L-Citrulline
L-Ornithine
L-Saccharopine
L-Tryptophan
Correlation
1
0.5
0
-0.5
-1
SrGene00568
SrGene03964
SrGene04265
SrGene04532
SrGene05419
SrGene05966
SrGene09200
SrGene09227
SrGene09735
SrGene10558
SrGene00484
SrGene01036
SrGene04584
SrGene05006
SrGene05264
SrGene07103
SrGene15342
SrGene03814
SrGene05183
SrGene09619
SrGene09066
SrGene09314
SrGene11195
SrGene05502
SrGene02315
SrGene04531
SrGene03057
SrGene01929
SrGene04166
SrGene05986
SrGene06984
SrGene09212
SrGene09568
SrGene02560
SrGene07100
SrGene07673
SrGene14664
SrGene04523
SrGene12841
SrGene01494
SrGene02604
SrGene04275
SrGene11578
SrGene12457
SrGene13436

(Figure S4)

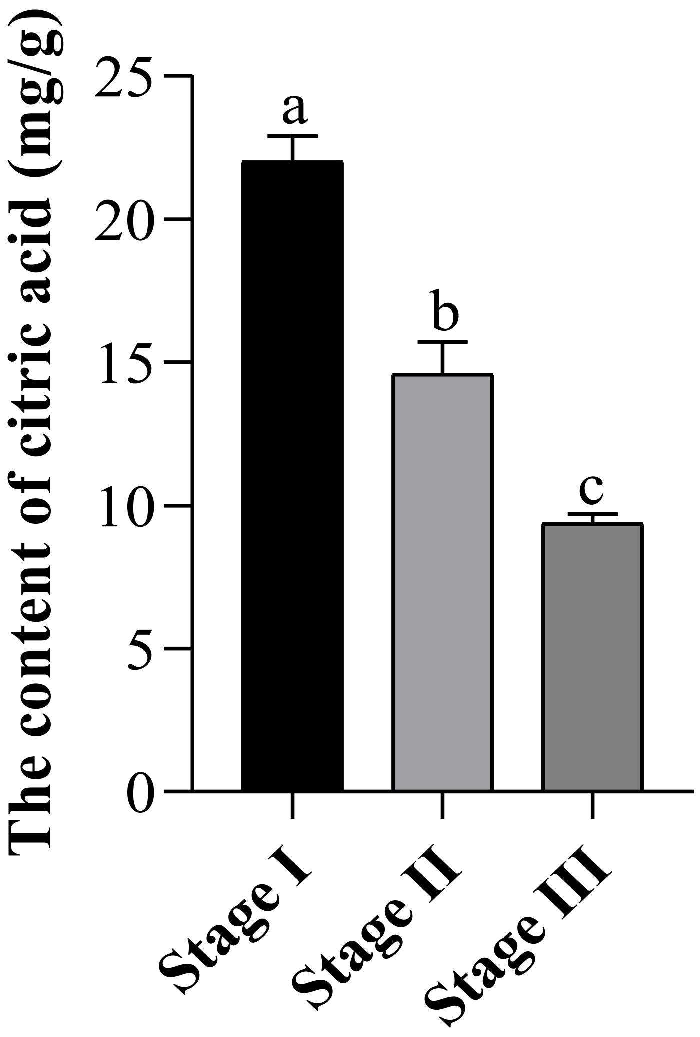
The content of citric acid (mg/g)
25
20
15
10
5
0
a
b
c
Stage I
Stage II
Stage III

(Figure S5)

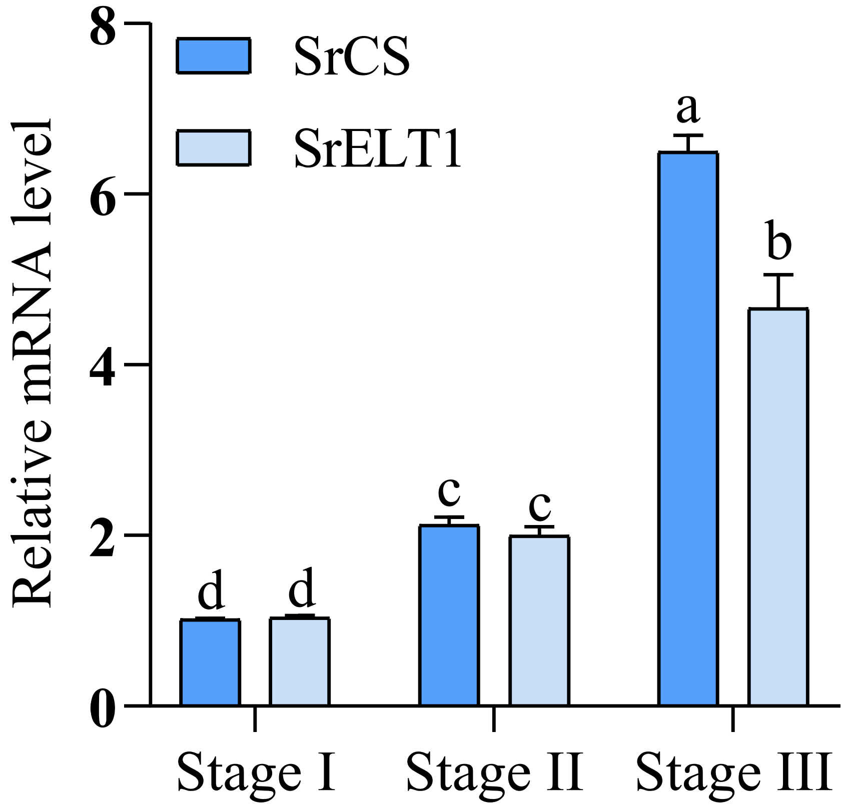

8
6
4
2
0
Relative mRNA level
SrCS
SrELT1
a
b
c
c
d
d
Stage I
Stage II
Stage III
